# Evaluating the influence of dental aesthetics on psychosocial well-being and self-esteem among students of the University of Novi Sad, Serbia: a cross-sectional study

**DOI:** 10.1186/s12903-024-04002-5

**Published:** 2024-02-26

**Authors:** Marija Stojilković, Ivana Gušić, Jelena Berić, Dušan Prodanović, Nevena Pecikozić, Tanja Veljović, Jelena Mirnić, Milanko Đurić

**Affiliations:** 1 Department of Dental Medicine, Faculty of Medicine, University of Novi Sad, Hajduk Veljkova 3, Novi Sad, 21000 Serbia; 2 Dentistry Clinic of Vojvodina, Hajduk Veljkova 12, Novi Sad, Novi Sad, 21000 Serbia; 3 Department of Pharmacology, Toxicology and Clinical Pharmacology, Faculty of Medicine, University of Novi Sad, Hajduk Veljkova 3, Novi Sad, 21000 Serbia

**Keywords:** Psychosocial impact of dental aesthetics questionnaire, Rosenberg self-esteem scale: students

## Abstract

**Background:**

A person’s smile has been identified as one of the first observed facial characteristics. Even minor deviations from societal beauty standards, especially among younger individuals, can have a negative effect on their self-esteem. The aim of this research is to evaluate the self-perceived psychosocial impact of dental aesthetics and self-esteem among respondents and their association, as well as to determine the main factors contributing to dissatisfaction with dental appearance.

**Methods:**

This research was conducted as a cross-sectional study that surveyed students of the University of Novi Sad. Other Universities and private faculties were excluded from participation. Data collection used standardized questionnaires measuring the Psychosocial Impact of Dental Aesthetics (PIDAQ) and the Rosenberg Self-Esteem Scale (RSES). Questionnaire (an online GoogleForms) was sent to the students via official Facebook groups of the faculties, student’s e-mails and Instagram profiles. Data analysis included descriptive statistics, Students T-test, ANOVA, multiple linear regression analysis and Spearman coefficient. To test internal consistency, Cronbach’s alpha(α) was calculated for the questionnaire as a whole (0,761) and each used questionnaire (PIDAQ – 0.766; RSES – 0.765). Cronbach’s alpha(α) was also calculated for each domain from PIDAQ (DSC-0.946; SI-0.882; PI–0.953; AC-0.916).

**Results:**

The study involved 410 participants, predominantly female (80%), aged between 21 and 23 (45.4%), primarily in ther first academic year (21.5%), and with a grade point average between 8.01 and 9.00 (42.4%). Data analysis has shown a statistically significant difference in the total PIDAQ score and SI subdomain in relation to the academic year (total PIDAQ *p* = 0.025; SI *p* = 0.000). In terms of self-esteem, results of multiple linear regression analysis showed that the academic year (95%CI: 0.410–1.837; *p* = 0.002) and the average point grade (95%CI: -0.025-1.600; *p* = 0.047) were significant predictors of greater self-esteem. The Spearman coefficient value (*r*=-0.316, *p* < 0.001) confirmed a statistically significant negative correlation between PIDAQ and self-esteem. Only 34% of respondents expressed satisfaction with their teeth. Dissatisfaction about the smile was primarily attributed to the arrangement and positioning of their teeth (32.2%). Financial constraints were identified as the main barrier for seeking interventions to improve smile satisfaction (39.5%).

**Conclusion:**

Students experiencing a greater psychosocial impact of dental aesthetics tended to exhibit lower self-esteem.

## Introduction

Physical appearance plays a pivotal role in the dynamics of social interaction. Among the facets constituting the overall appearance, facial features are of paramount significance, where the eyes and mouth are particularly important as they are the primary focal points for interaction and communication [1]. A person’s smile has been identified as one of the first observed facial characteristics, with overall facial attractiveness determined within a few seconds [2]. Notably, the orofacial region garners substantial attention during interpersonal interactions and is the primary source of vocal, physical, and emotional communication [3].

In contemporary society, an inherent need for acceptance exists. Social norms established by friends, peers, and social networks impact individuals significantly [4, 5]. As a result, even minor deviations from societal beauty standards, especially among younger individuals, can have a negative effect on their self-assurance and self-esteem, ultimately influencing their overall quality of life [6]. Previous researches have shown a correlation between negative evaluations of one’s body and appearance with the lack of self-esteem and adverse mental health outcomes such as anxiety and depression [7–9]. Numerous studies have examined the influence of oral health on the quality of life among young individuals, highlighting the significant role played by dental aesthetics. It has been noted that even minor irregularities in tooth appearance can impact students’ oral health-related quality of life. This is manifested in concerns regarding social presentation, dissatisfaction with one’s appearance, and a diminished sense of self-esteem [6, 10–12].

Nathaniel Branden defines self-esteem as confidence in our right to be happy, the feeling of being worthy, deserving, and entitled to assert our needs and wants and enjoy the fruits of our efforts [13]. It is an essential requirement for humans, because it helps them to stay motivated and feel good about themselves. In addition, it boosts the morale of human beings by building a positive self-image and attitude [14]. Higher self-esteem contributes to easier coping in social interactions, empowering individuals to assert themselves; people are more ready to speak loudly but also offer constructive critique, manage challenges efficiently, and propose alternative solutions [15]. Self-esteem in younger populations, such as college students, has been proven to be of utter importance as they undergo transitions and identity changes that require a strong sense of self [16]. During this period, individuals embark on a journey to enhance self-worth, embrace self-care practices, and solidify their social identity, all while grappling with the objective evaluation of their appearance [17].

According to the World Health Organization, health goes beyond the absence of illness. It includes being physically, mentally, and socially well. Having this in mind, it is essential to closely examine how dental aesthetics affect a person’s psychological and social well-being, as well as their self-esteem. One of the most widely used instruments in the evaluation of the dental aesthetics’ psychosocial is the Psychosocial Impact of Dental Aesthetics Questionnaire (PIDAQ) [18]. The Rosenberg self-esteem (RSES) scale stand out as a prevalent and extensively used measure of self-esteem [19].

The PIDAQ was created by Klages et al. to assess self-perception of dental aesthetics. It consists of a total of 23 questions divided into four subdomains. The first part of the questionnaire is Dental Self-Confidence, which measures a positive dental body concept. The second part contains questions about Social Impact, which examine possible fears and problems an individual may experience in social interactions due to the appearance of their teeth. The third part includes questions about Psychological Impact, which evaluates feelings of inferiority or unhappiness related to an individual’s comparing self with others. The last part of the questionnaire is Aesthetic Concern, which covers concerns about the appearance of teeth when an individual looks in the mirror or sees themselves in photographs or videos [18].

Several questionnaires measure self-esteem, but the Rosenberg Self-Esteem Scale is the most commonly used due to its simplicity, brevity, and ease of understanding for respondents. Developed by Rosenberg in 1965, it has a long history of use. It is a one-dimensional questionnaire focused on an individual’s self-esteem regarding their own worth. It consists of 10 questions, with five posed in a positive and 5 in a negative context [19].

To date, no study has examined the psychosocial impact of dental aesthetics and its correlation with self-esteem among University of Novi Sad students. Thus, this research evaluated the self-perceived psychosocial impact of dental aesthetics and self-esteem among respondents and their association. The research posits the hypothesis that individuals experiencing substantial psychosocial effects due to dental aesthetics are likely to exhibit lower levels of self-esteem.

## Materials and methods

### Study design and participants

The conducted research was a cross-sectional study that surveyed students of the University of Novi Sad. The data collection phase extended over a span of three months, from December 2022 to March 2023. The inclusion criteria comprised the University of Novi Sad students, excluding students from other universities and private faculties in Novi Sad due to their limited representation in Novi Sad.

### Sampling and sample size

A minimum sample size of 381 respondents was calculated based on the margin of error (5%), confidence level (95%), response distribution (50%), and the population size of students at the University of Novi Sad (≈ 40,000) [20].

Participants were selected through convenience sampling method.

### Survey Design

Data collection used standardized questionnaires measuring the Psychosocial Impact of Dental Aesthetics (PIDAQ) and the Rosenberg Self-Esteem Scale (RSES).

The PIDAQ is a psychometric instrument with 23 questions divided into four domains. The first part of the questionnaire examined Dental Self-Confidence (DSC) and included the first six questions, whereas, the second part examined Social Impact (SI) and included questions 7 to 14. The third part examined Psychological Impact (PI) and included questions 15–20, and the fourth part and final part about Aesthetic Concern (AC) included the last three questions. The questions from the first part of the questionnaire were asked in the positive, while all other questions were asked in the negative.

The RSES is a psychometric instrument consisting of 10 questions, five being positive and five being negative (questions 2, 5, 6, 8, and 9 are negative). The respondents answered both questionnaires using a 5-point Likert scale, with numerical values 1 representing “Strongly disagree”, 2 “I do not agree“, 3 “I am not sure”, 4 “I agree” and 5 “Strongly agree”.

In addition to PIDAQ and RSES items, the survey included sociodemografic variables such as gender, age, current academic year, and grade point average. The participants were also asked to select specific smile components for dissatisfaction with the smile.

### Development of a translated version of PIDAQ

#### Translation

The PIDAQ and RSES were translated by two independent translators, one native English speaker who is fluent in Serbian and one native Serbian speaker who is fluent in English. Both of them were familiar with dental and Quality of Life terminology and instruments.

#### Back translation

An English teacher, unaware of the content of the original English questionnaire, conducted a back-translation of the Serbian version of the PIDAQ into English.

#### Committee review

A „double-blind“evaluation of the translated versions was implemented with regard to the translator and the back translator. The original and translated versions were compared by a committee of two specialists in Periodontology and Oral Medicine and one specialist in Psychiatry, all fluent in English and familiar with Quality of Life tools. The committee evaluated whether words in both the original and adapted questionnaire versions conveyed the same idea. Adjustments to the instrument were made based on the consensus of the committee members.

After semantic and conceptual equivalence were assessed and compared with the original questionnaire, the first Serbian version of the PIDAQ was produced, after which the pilot research was conducted.

The pilot was tested on a convenience sample of 30 students from Novi Sad University who evaluated the appropriateness of the questions. The responses from those participants were not included in the study. A few linguistic modifications and a final semantic adjustment were made according to their comments. The pilot analysis demonstrated that the University of Novi Sad students could easily understand the questionnaire. Therefore, the cross-cultural adaptation resulted in a tool ready to be sent to the participants.

### Validity of questionnaire

The expert committee established content and face validity with a Content Validity Index (CVI) 0.94. Further review of the literature also confirmed the face validity.

Construct validity for PIDAQ was assessed using factor analysis. The Kaiser‑Meyer‑Olkin measure of sampling adequacy was 0.956, and Bartlett’s test of sphericity was 8668.7 (*p* < 0.001). These results showed that the variables were within the normal range and appropriate for inclusion in factor analysis. The exploratory factor analysis detected three factors with an Eigenvalue greater than 1.0, with the item factor loadings ranging from 0.360 to 0.803. The first extracted domain contained items 1–6, comprising the Dental Self-Confidence (DSC) subscale, and explained 44.91% of the variance. The second extracted domain contained Social Impact subscale items 7–14 and explained 12.44% of the variance. The third extracted domain contained items 15–23, representing the Psychological Impact subscale, and explained 8.86% of the variance. In total, these three components explained 66.21% of the total variance.

RSES items were factor analyzed using exploratory factor analysis. The Kaiser-Meyer-Olkin measure of sampling adequacy was 0.893, and Bartlett’s test of sphericity was 2209.123 (*p* < 0.001), showing that RSES items could be factorized. Factor analysis detected two factors with the item factor loadings ranging from 0.556 to 0.868. The first extracted domain contained items in a negative context and explained 46.65% of the variance, while the second extracted domain contained items in a positive and explained 14.45% of the variance. These two domains explained the total variance of 61.11%.

### Reliability of questionnaire

To test internal consistency, Cronbach’s alpha (α) was calculated for the questionnaire as a whole (0.761) and each used questionnaire (PIDAQ – 0.766; RSES – 0.765). Cronbach’s alpha (α) was also calculated for each domain from PIDAQ (DSC (6 items) – 0.946; SI (8 items) – 0.882; PI (6 items) – 0.953; AC (3 items) – 0.916). The scale was considered reliable when Cronbach’s alpha (α) was more than 0.7, thus indicating acceptable to excellent internal consistency. If the item was deleted, the values of Cronbach’s alpha revealed that excluding an item from PIDAQ and RSES questionnaires analysis did not significantly improve Cronbach’s Alpha, reinforcing the indication of strong internal consistency.

### Questionnaire distribution

The final version of the questionnaire (in an online GoogleForm) was distributed to the students via official Facebook groups of the faculties, student’s e-mails and Instagram profiles. Alongside the questionnaire, a cover letter was mailed, as well, which informed the participants about the study aims and participant confidentiality. The participation in the research was voluntary without financial compensation. They were allowed to withdraw at any time. The research method, through Google’s privacy policy, guaranteed the anonymity of the respondents.

### Statistical analysis

The psychosocial impact of dental aesthetics was considered an outcome variable (PIDAQ), which was calculated by summing the total scores of the subscales SI, PI, AC and reversed scores of the positive domain DSC. The possible range of the PIDAQ score was 23–115. A low PIDAQ score indicated a low psychosocial impact of dental aesthetics, whereas a high score indicated a high psychosocial impact. The total PIDAQ score was dichotomized based on the median into low and high impacts (with a value of 1 assigned if the respondents had a high phychosocial impact of dental aesthetics, and 0 if it was low). Binary logistic regression analysis was used to determine whether independent variables (gender, age, academic year and grade point average) were associated with the PIDAQ score (dependent variable).

The total self-esteem score was calculated by summing the items of the RSES questionnaire, after reverse coding the relevant items (questions 2, 5, 6, 8 and 9). The possible range of the score was 10–50. Low RSES score indicated low self-esteem, whereas high score indicated high self-esteem. Multiple linear regression was used to determine whether independent variables (gender, age, academic year and grade point average) were associated with self-esteem score (dependent variable).

Descriptive statistics (means, standard deviation and frequencies) were used to describe the sociodemographics characteristics and the mean of the PIDAQ and RSES scores. Student’s t-test and bivariate analysis (ANOVA) were used to compare the mean differences in various aspects, including the overall psychosocial impact of dental aesthetics, its individual subscales (dental self-confidence, social impact, psychological impact and aesthetic concern), as well as the total self-esteem score concerning sociodemographics characteristics (gender, age, year of study, average point grade).

Spearman’s correlation coefficient was used to assess the correlation between the psychosocial impact of dental aesthetics and self-esteem.

Statistical analyses were performed using SPSS Statistics for Windows version 24 (IBM Corporation, Armonk, NY, USA). The results were evaluated within a 95% CI. *P* values that were < 0.05 were considered statistically significant.

### Ethical aspects of the research

The study was conducted according to the guidelines of the Declaration of Helsinki. Ethical approval for the research was obtained from the Ethics Committee of the Faculty of Medicine Novi Sad, Serbia (No.: 01–39/298/1). Every survey included Informed Consent Statement, in which the participants were assured about confidentiality of their responses by using anonymous questionnaire. In addition, they were informed that their participation was voluntary and that they could stop filling out the questionnaire at any time without any consequences.

## Results

Of the 550 questionnaires distributed, 410 participants completed the questionnaire, resulting in a response rate of 74.54%. The distribution of sociodemographic characteristic are presented in Table 1. In terms of gender distribution, most of the participants were female (80%). A higher percentage of the respondents were between the ages of 21 and 23 (45.4%), in their first academic year (21.5%) and with a grade point average between 8.01 and 9.00 (42.4%).

**Table 1:** Sociodemographic characteristics of the respondents (*n* = 410)

	n		%
Total	410		100
Gender			
Male	82		20
Female	328		80
Age			
18–20	128		31.2
21–23	186		45.4
> 24	96		23.4
Academic year			
First	88		21.5
Second	47		11.5
Third	78		19
Fourth	70		17.1
Fifth/sixth	70		17.1
Master/PhD	57		13.8
Grade point average			
6.00–7.00	14		3.4
7.01–8.00	87		21.2
8.01–9.00	174		42.4
9.01–10.00	135		33

Overall, the mean for the total PIDAQ score was 50.68 (SD = 20.91). In terms of the specific domain of the questionnaire, the highest rating was given to Dental Self-Confidence (DSC) domain (Mean = 15.05, SD = ± 6.64), followed by Psychological Impact (PI) domain (Mean = 14.46, SD = ± 6.05), Social Impact (SI) domain (Mean = 14.13, SD = ± 7.41) and Aesthetic Concern (AC) domain (Mean = 7.02, SD = ± 3.59) (Table 2).

**Table 2:** Descriptive statistics for PIDAQ scales

	*n* = 410				
	Minimum	Maximum	Median	Mean	SD
**Total PIDAQ**	23	115	47.00	50.68	± 20.91
**DSC**	6	30	14.00	15.05	± 6.64
**SI**	8	40	11.00	14.13	± 7.41
**PI**	6	30	14.00	14.46	± 6.05
**AC**	3	15	6.00	7.02	± 3.59

The results showed that there was no statistically significant difference in the total PIDAQ score with regard to gender, age and grade point average. However, a statistically significant difference was observed in to relation academic year. Specifically, participants in their first academic year showed a more pronounced psychosocial impact of dental aesthetics compared to those in more advanced academic years (*p* = 0.025). Regarding the PIDAQ subscales, there was no statistically significant difference in Dental Self-Confidence, Psychological Impact, and Aesthetic Concern with regard to any of the sociodemographic variables. However, a statistically significant difference was found in the Social Impact concerning academic year. The participants in their first academic year showed that dental aesthetics had a greater social impact on them compared to those enrolled in more advanced academic years (*p* = 0.000). (Table 3)

**Table 3:** Comparison of the mean total PIDAQ score and subscale scores among different sociodemographic variables

Sociodemographic variables		Total PIDAQ (Mean ± SD)	p	DSC (Mean ± SD)	p	SI (Mean ± SD)	p	PI (Mean ± SD)	p	AC (Mean ± SD)	p
Gender	Male	49.75 ± 20.31	0.487	15.31 ± 6.59	0.749	13.73 ± 7.56	0.911	14.06 ± 6.13	0.966	6.64 ± 3.36	0.323
	Female	50.91 ± 21.08		14.99 ± 6.66		14.23 ± 7.38		14.56 ± 6.03		7.11 ± 3.64	
Age	18–20	52.05 ± 21.52	0.581	15.45 ± 6.62	0.470	15.00 ± 8.07	0.166	14.57 ± 6.33	0.280	7.01 ± 3.61	0.757
	21–23	49.22 ± 20.25		14.48 ± 6.50		13.55 ± 6.84		14.12 ± 5.74		7.04 ± 3.64	
	> 24	51.67 ± 21.36		15.63 ± 6.92		14.08 ± 7.52		14.96 ± 6.27		6.98 ± 3.44	
Academic year	First	55.60 ± 22.49	0.025*	15.09 ± 6.80	0.706	15.85 ± 7.98	0.000*	14.50 ± 6.58	0.448	6.88 ± 3.70	0.642
	Second	47.46 ± 17.94		14.08 ± 5.95		13.25 ± 6.10		13.57 ± 5.20		6.55 ± 3.50	
	Third	51.68 ± 22.42		16.06 ± 6.63		15.20 ± 8.29		15.78 ± 6.19		7.89 ± 3.55	
	Fourth	48.88 ± 21.08		14.98 ± 6.55		13.54 ± 7.57		13.61 ± 5.75		6.74 ± 3.72	
	Fifth/sixth	45.35 ± 16.55		13.97 ± 6.59		15.33 ± 7.87		13.71 ± 5.86		6.58 ± 3.33	
	Master/PhD	53.80 ± 21.73		15.84 ± 7.07		11.07 ± 4.35		15.13 ± 6.03		7.31 ± 3.62	
Grade point average	6.00–7.00	50.00 ± 14.79	0.169	16.00 ± 7.93	0.602	14.21 ± 6.77	0.734	13.85 ± 4.94	0.414	5.92 ± 3.02	0.363
	7.01–8.00	51.58 ± 20.87		15.03 ± 6.83		14.50 ± 7.26		15.08 ± 5.68		6.96 ± 3.41	
	8.01–9.00	49.24 ± 21.88		14.85 ± 6.88		13.63 ± 7.35		13.81 ± 6.23		6.93 ± 3.67	
	9.01–10.00	52.02 ± 20.25		15.22 ± 6.10		14.52 ± 7.69		14.97 ± 6.11		7.28 ± 3.65	

The p-value was calculated using t-test and ANOVA for categorical variables. A statistically significant difference at *p* < 0.05.

The results have shown no statistically significant difference in self-esteem with regard to gender and age. However, a statistically significant difference in relation to the year of study was discovered, where respondents pursuing Master’s and PhD programs exhibited the highest self-esteem (*p* = 0.001). Furthermore, a statistically significant difference was observed in relation to the participants’ average grades during their studies. Respondents with an average score within the range of 9.01 to 10.00 have greater self-esteem than those with lower average scores (*p* = 0.002). (Table 4)

**Table 4:** Rosenberg’s self-esteem scale in relation to different sociodemographic variables

	Mean ± SD	p
Gender		
Male	32.62 ± 6.71	0.974
Female	32.88 ± 6.89	
Age		
18–20	31.40 ± 7.46	0.283
21–23	33.61 ± 6.51	
> 24	33.21 ± 6.85	
Academic year		
First	31.10 ± 7.41	0.001*
Second	32.82 ± 7.13	
Third	31.00 ± 6.75	
Fourth	34.28 ± 6.33	
Fifth/sixth	34.14 ± 6.45	
Master/PhD	34.52 ± 6.01	
Grade point average		
6.00–7.00	27.28 ± 7.04	0.002*
7.01–8.00	31.17 ± 6.20	
8.01–9.00	33.60 ± 6.82	
9.01–10.00	33.64 ± 6.85	

The *p*-value was calculated using t-test and ANOVA for categorical variables. A statistically significant difference at *p* < 0.05.

Multivariate binary regression results did not show any correlaton between PIDAQ scores and sociodemographic characteristics in any of the all four domains.

The results of multiple linear regression analysis showed that academic year (95% CI: 0.410–1.837; *p* = 0.002) and average point grade (95% CI: -0.025–1.600; *p* = 0.047) were significant predictors of greater self-esteem. (Table 5)

**Table 5:** Multiple linear logistic regression model; Rosenberg’s self-esteem scale in relation to different sociodemographic variables

	Unstandardized Coefficient		Standardized Coefficients	95% CI		Sig.
	B	SE	Beta	Lower	Upper	
Constant	29.761	2.081		25.669	33.852	0.000
Gender	− 0.360	0.842	− 0.021	-2.016	1.295	0.669
Age	-1.293	0.828	− 0.139	-2.921	0.336	0.119
Academic year	1.124	0.363	0.281	0.410	1.837	0.002*
Grade point average	0.788	0.413	0.095	− 0.025	1.600	0.047*

B—regression coefficient; SE—standard error; CI—Confidence Interval OR—Odds Ratio

**Fig. 1:**
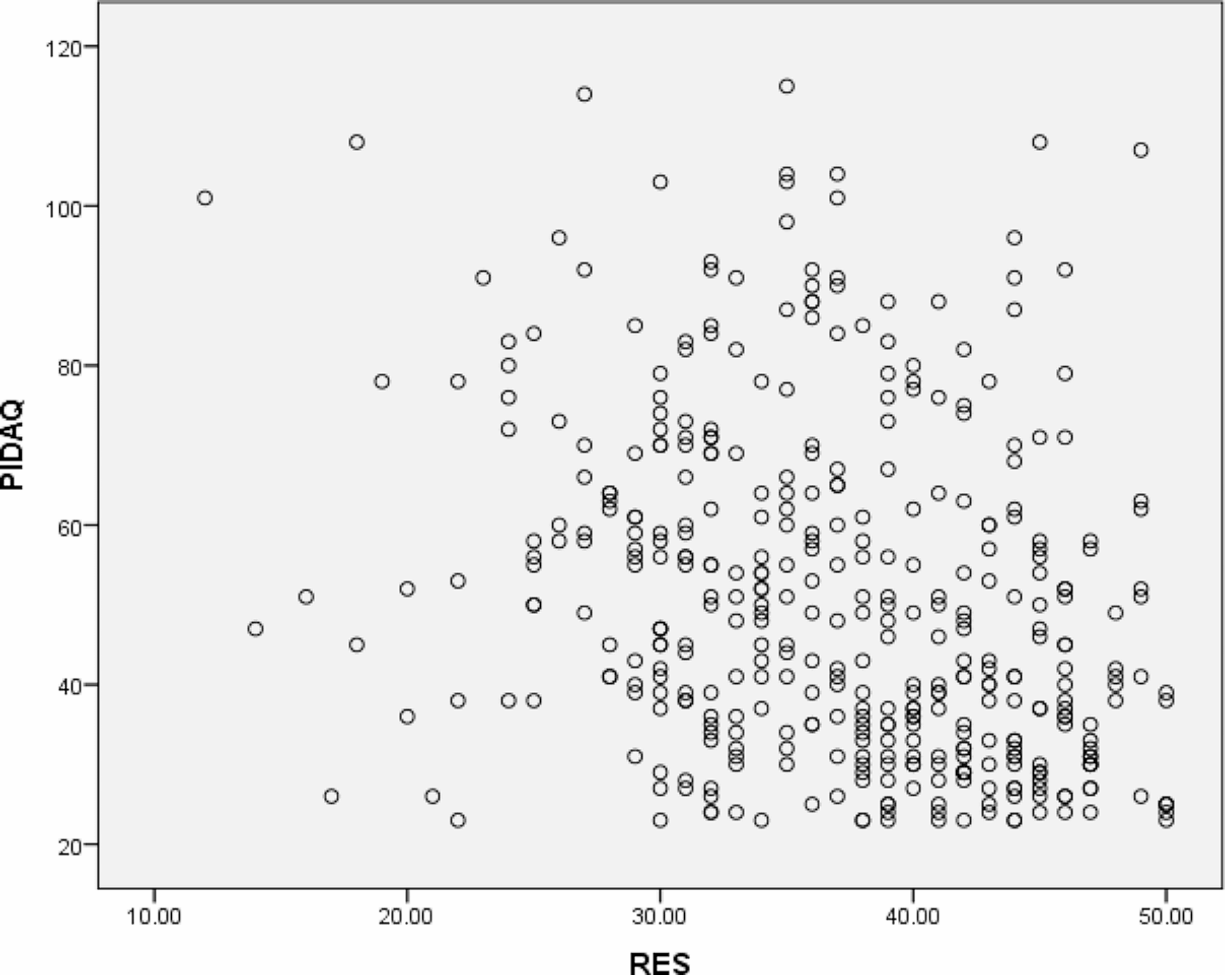
Scatter plot of correlation between PIDAQ and RSES

Using Spearman’s correlation coefficient, we investigated the correlation between the psychosocial impact of dental aesthetics and self-esteem. The Spearman coefficient value (*r* = -0.316, *p* < 0.001) has confirmed a statistically significant negative correlation. Specifically, participants who were more affected by the psychosocial impact of dental aesthetics tended to have lower self-esteem. (Table 6; Fig. 1).

**Table 6:** Spearman correlation coefficient of psychosocial impact of dental aesthetics and self-esteem

Spearman’s correlation coefficient		PIDAQ	RES
PIDAQ	Correlation coefficient	1,000	-0,324*
	Statistical significance		**0,000**
	N	410	410
RES	Correlation coefficient	-0,324*	1,000
	Statistical significance	**0,000**	
	N	410	410

**. The correlation is statistically significant at the 0.01 level (2-tailed)

The largest number of the respondents were satisfied with their teeth (34%). Among those who expressed dissatisfaction, the most prevalent cause was related to the arrangement and positioning of their teeth (32.2%). In addition, 34.6% of the respondents said that orthodontic treatment was a potential solution to enhance their smile satisfaction. The main reason why respondents did not perform any needed interventions in order to be more satisfied with their smile was finances (39.5%). (Table 7)

**Table 7:** Respondents’ opinions about the appearance of their teeth

**If you are not satisfied with your teeth, what are the main reason for that?**		
Tooth color	78	19
Tooth size	13	3.2
Arrangement and positioning of the teeth	132	32.2
Gum color and position	26	6.3
Other	22	5.3
I am satisfied with my teeth	139	34
**Which of the following dental interventions would help you be more satisfied with the appearance of your teeth?**		
Orthodontic therapy	142	34.6
Prosthetic therapy	41	10
Resto therapy	36	8.8
Calculus and pigmentation removal	73	17.8
Gum’s position correction	26	6.4
Other	92	22.4
**What is the main reason why you haven’t done the intervention you think you need so far?**		
Fear of the dentist	45	11
Financial reason	162	39.5
Lack of time	45	11
Concern about the outcome of the intervention	42	10.2
Other	116	28.3

## Discusion

The modern world’s emphasis on appearance, fueled by the rise of social media and constant exposure to idealized images, has given rise to a culture in which the pursuit of aesthetic perfection can carry significant weight [21, 22]. Dental aesthetics, as an integral aspect of one’s overall appearance, is no exception. A dazzling smile is often equated with confidence, success, and attractiveness [23].

The mean PIDAQ score obtained for the study sample was 50.68 ± 20.91. When compared with previous studies, including those involving a similar population sample, this score is considered low, suggesting a low psychosocial impact related to dental aesthetics among the University of Novi Sad students [24–26].

In comparison with the sociodemographic characteristics, a statistically significant difference was observed in relation to the years of study. Specifically, the respondents in their first year of study showed that dental aesthetics had the greatest psychosocial impact on them. College life exposes students to a diverse range of individuals. This can lead to an increased tendency to compare themselves with others, which may drive the desire to conform to certain beauty ideals. For example, having a bright and even smile might be a way for them to fit into what society sees as attractive [2]. Moreover, our findings are in contrast to those by Alsagob et al., in which dental esthetics had the most pronounced impact on senior students [5].

Furthermore, our results indicate that there was no statistically significant difference in the psychosocial impact of dental aesthetics between genders, which is in agreement to the results obtained by Ellakany et al. after their research in Saudi Arabia [27]. The researches that have been exploring the association between aesthetics and gender consistently show that women tend to have higher expectations regarding beauty and aesthetics, as a result of them being sensitive to what they perceive as flaws in their facial and bodily appearance [28, 29]. However, it is important to highlight that a statistically significant difference in gender was observed in our research, with a prevalence of female students. This factor could potentially impact the obtained results. Our findings also indicate that there were no statistically significant differences in the psychosocial impact of dental aesthetics based on age or the average grade point.

The results of our research also showed that there was no statistically significant difference in dental self-confidence, psychological impact, and aesthetic concern regarding any of the sociodemographic characteristics. Nevertheless, the results did reveal a statistically significant difference in the social impact of dental aesthetics among the respondents from varying years of study. Specifically, the participants in their first year of the studies reported a significantly greater social impact of dental aesthetics compared with those in other academic years. Our hypotheses that the transition from high school to college is a pivotal phase of life, marked by the challenge of adapting to unfamiliar surroundings. During this life period, students not only adjust to academic demands but also navigate new social connections. Their heightened awareness of appearance and the desire to make a positive impression on peers can amplify the importance of dental aesthetics during transition. Individuals dissatisfied with their teeth or smile may feel self-conscious in social situations. This can cause them to develop negative coping mechanisms, such as avoiding smiling or social settings altogether. As a result, they may struggle to develop strong interpersonal and social skills, which can impact their ability to adapt to society. Rai et al. [30] did not find any influence of the education level of psychosocial impact of dental aesthetics. However, they observed that respondents with higher education showed better psychological adaptation to dental appearance, which was attributed to the notion that a higher educational level is associated with reduced self-consciousness. This phenomenon could be explained by the fact that individuals with higher education levels typically engage in more social interactions and tend to develop greater self-confidence compared to those with lower levels of education.

A statistically significant difference was observed in self-esteem with respect to the academic year of study and the average grade attained at the University. The results of multiple linear logistic regression analysis indicated that higher academic programs and higher grade point averages are significant predictors of greater self-esteem. The connection between academic achievement and self-esteem is open to various interpretations. One perspective proposes that achieving good grades automatically improves students’ self-perception, nurturing a sense of self-worth and directly boosting their self-esteem. Conversely, certain researches suggests a potential influence of self-esteem on academic performance. This viewpoint has spurred the development of diverse educational programs and interventions to enhance students’ grades by bolstering their self-esteem. Numerous studies have identified a significant correlation between academic performance and self-esteem [31]. However, it is essential to emphasize that correlation does not imply causation. The reciprocal interplay between self-esteem and academic achievement continues to be a compelling subject for exploration and investigation. It provokes a valuable inquiry into whether self-esteem molds academic achievement or, conversely, if academic achievement shapes self-esteem.

The outcomes of our research indicate that there is no statistically significant difference in self-esteem with respect to age and gender. However, the studies conducted thus far have indicated contradictory findings. For instance, Mohammad et al. [32] noted that females demonstrated higher levels of self-esteem, whereas in a study by Muhammad Ahsan [33], male students exhibited higher self-esteem in comparison to females. Our findings are consistent with those of Asif Amin et al. [34] who did not find significant difference among male and female students in terms of self-esteem. We reiterate that the significantly higher number of female students in this research may impact obtained results.

The present study observed a negative correlation between the psychosocial impact of dental aesthetics and self-esteem. This implies that the individuals who are more influenced by dental aesthetics tend to have lower self-esteem. Similar findings were reported by Venete el at [35]. who found the positive correlation between self-esteem and PIDAQ Self-Confidence subdomain. In other words, dental satisfaction has a positive effect on self-esteem. Akpasa et al. [36] revealed that the self-perception of smile and dental aesthetics is a significant factor that influences self-esteem among adolescents, as well. In addition, several studies have highlighted that the individuals may be judged on the basis of their dental aesthetics. For example, poor dental aesthetics have been associated with diminished perception of intelligence. Consequently, individuals with more of a pleasing smile have a higher chance of getting a job and enjoying a better quality of life [37–39].

Key factors influencing the overall look of teeth include their color, shape, alignment, and arrangement, especially regarding those at the front. Additionally, a smile that is considered aesthetically pleasing depends on all those factors, together with the position of the upper lip, how many of the teeth can be seen and how much of the gums are visible [40, 41]. The results of the present study revealed that only 34% of the respondents were satisfied with their smile, which is a notably lower percentage compared to the study conducted by Ellkany et al. [27] among students in Saudi Arabia. In their study, the primary reasons for smile dissatisfaction were related to teeth alignment and color, which is consistent with our research. In our study, 32.3% of respondents pointed to the arrangement and positioning of their teeth as the primary reason for their dissatisfaction with their smile, while 19% identified teeth color as a significant factor. Additionally, thirty-four respondents from our study believed that orthodontic therapy would help them to be more satisfied with their smile. The primary obstacle preventing respondents from pursuing interventions they believed were necessary to enhance their smile satisfaction was financial constraints, as reported by 39.5% of the respondents. Unfortunately, the data about respondents’ monthly income was not collected during this research, thus preventing us to assess whether there is a statistically significant difference in relation to this variable.

The study benefits from the utilization of standardized questionnaires, allowing comparison and discussion with many other studies. Homogenizing the group of respondents to students only from the University of Novi Sad provided a clearer insight into the cultural and social influences of the environment. On interpreting the results of the present study, it is important to highlight its limitations. Firstly, a notable limitation of the study lies in the use of self-perceived dental aesthetics as a subjective measure. Furthermore, it is important to note that the study has limitations related to the distribution of genders. The difference in the number of male and female participants could potentially influence the obtained results, even though there is a higher number of female students at the University of Novi Sad. Additionally, in order to generalize the obtained results to the entire student population of the University of Novi Sad, a larger sample would be desirable. In this study, students from private colleges were excluded due to the limited number of private colleges in Novi Sad and, consequently, a small number of students. More detailed and comprehensive results could be obtained by including sufficient students from private colleges. Moreover, perceived dental aesthetics are influenced by multiple factors. Unexplored variables such as socio-economic status, lifestyle, ongoing aesthetic treatments and expectation of the treatment outcome could be opportunities for future research.

## Conclusion

Dental aesthetics had the greatest psychosocial impact on the first-year of study, whereas there was no difference in the comparison by gender, age, and average grade point during the study. Notably, those in Master’s and PhD programs demonstrated higher self-esteem compared to those with fewer years of study and a lower average point. Respondents with a heightened psychosocial impact related to dental aesthetics tended to have lower self-esteem. The majority of respondents expressed dissatisfaction with the position and arrangement of their teeth and believed orthodontic therapy would improve their satisfaction.

## Data Availability

The datasets used and analyzed during the current study are available from the corresponding author upon reasonable request.
